# Long-term outcome of percutaneous vertebroplasty versus conservative treatment for osteoporotic vertebral compression fractures: a retrospective cohort study with three-year follow-up

**DOI:** 10.3389/fmed.2024.1391243

**Published:** 2024-05-03

**Authors:** Zefu Chen, Lei Xu, Liang Shi, HongXia Cao, Mingxi Nie

**Affiliations:** ^1^Department of Emergency, Xiangyang No.1 People's Hospital, Hubei University of Medicine, Xiangyang, China; ^2^Department of Orthopedics, Xiangyang No.1 People's Hospital, Hubei University of Medicine, Xiangyang, China; ^3^Department of Rehabilitation Medicine, Xiangyang No.1 People’s Hospital, Hubei University of Medicine, Xiangyang, China

**Keywords:** osteoporotic vertebral compression fractures, percutaneous vertebroplasty, conservative treatment, long-term outcomes, complications, satisfaction rate, new vertebral compression fractures

## Abstract

**Background:**

Osteoporotic vertebral compression fractures (OVCF) appear to be more common as the population ages. Previous studies have found that percutaneous vertebroplasty (PVP) can achieve better short-term clinical outcomes than conservative treatment (CT) for OVCF. However, the long-term outcomes of PVP compared with CT for OVCF has been rare explored. This study was designed to explore the clinical outcomes of PVP or CT within 3 years after OVCF.

**Methods:**

This study reviewed the clinical outcomes of patients who underwent PVP or CT for OVCF in a single center from January 2015 to December 2019. The back pain visual analogue scale (VAS), Oswestry disability index (ODI) and satisfaction rate were compared between the two groups at baseline, 1 week, 1 month, 3 months, 6 months, 12 months, 24 months and 36 months after treatment.

**Outcomes:**

The baseline data including gender, age, bone mineral density, body mass index, back pain VAS, and ODI were not significantly different between the two groups. The back pain VAS and ODI of CT patients were significantly higher than those of PVP group at 1 week, 1 month, 3 months, 6 months and 12 months after treatment. The satisfaction rate in the PVP group were significantly higher than those in the CT group at 1 week, 1 month, 3 months and 6 months after treatment. Subsequently, the back pain VAS and ODI showed no significant difference between the two groups at 24 and 36 months. In addition, there was no significant difference in treatment satisfaction between the two groups at 36 months. There was no significant difference in the rate of new vertebral compression fractures between the two groups within 36 months after treatment.

**Conclusion:**

The clinical outcomes within 12 months after PVP and patient satisfaction rate within 6 months after PVP were significantly higher than CT. However, during 12 months to 36 months, this advantage generated by PVP was gradually diluted over time. Compared with CT, the long-term effect of PVP on OVCF should not be overestimated.

## Introduction

With the inevitable increase in aging population and the improvement in life expectancy, age-related diseases such as osteoporosis is becoming more common in the population (1, 2). In the United States, osteoporosis has been reported in 16% of men and 29.9% of women over the age of 50 years (3). In China, the prevalence of osteoporosis is 10.4% in men and 31.2% in women over 50 years of age (4). As age increases, patients with osteoporosis are more prone to osteoporotic vertebral compression fractures (OVCF) (5, 6). OVCF is usually accompanied by persistent severe back or referred pain, which severely limits the patient’s quality of life (5, 6).

Generally, OVCF can be treated conservatively or surgically (7). Before the advent of percutaneous vertebroplasty (PVP), conservative treatment (CT) for OVCF was the first-line treatment. However, the use of PVP has changed this treatment choice (8–10). PVP may provide better or faster pain relief or improve disability compared to CT (11–18). However, previous studies have focused on early and intermediate clinical outcomes (usually within 12 months) (11–15, 17, 18), and few have compared the long-term clinical outcomes of patients treated with these two treatment modalities (16). In addition, patient satisfaction rates with PVP or CT for OVCF have rarely been studied previously (15). Therefore, this study aimed to explore the short-and long-term clinical outcomes and satisfaction rates of patients with OVCF treated with PVP or CT to provide relevant guidance for the treatment of patients with OVCF.

## Methods

This study retrospectively analyzed the clinical and imaging data of patients with OVCF who underwent PVP or CT at a single spine center between January 2015 and December 2019. The study was reviewed and approved by the hospital ethics committee and all patients signed the informed consent form. All the authors adhered to the Declaration of Helsinki (2013) in this study. All the methods meet the requirements of ethical, moral and scientific principles.

Inclusion criteria:

(1) Patients who underwent PVP or CT for OVCF. (2) Patients who were followed up for at least 36 months. (3) Patients with complete clinical data.

Exclusion criteria:

(1) Patients with other severe systemic diseases such as myocardial infarction, cerebral infarction, cerebral hemorrhage, Parkinson’s syndrome, Alzheimer’s disease, or accompanied by nauseous tumors. (2) Patients who were lost to follow-up or died during follow-up. (3) Patients who could not complete the follow-up due to personal reasons.

### Collection of demographic data

Demographic and clinical outcome data were collected from the hospital’s inpatient cases system. A qualified clinical follow-up staff collected data on diagnosis, treatment methods, sex, age, bone mineral density (BMD), body mass index (BMI), and history of accompanying diseases. In addition, patients who experienced new vertebral compression fractures (NVCF) within 36 months were recorded and NVCF rates were calculated in both groups. The diagnosis of NVCF was determined after the patient experiences PVP or CT, followed by a sudden onset of severe back pain and the presence of a new vertebral fracture confirmed by magnetic resonance imaging (MRI).

### Evaluation of clinical outcomes

The patients’ back pain visual analogue scale (VAS) (19) and Oswestry Disability Index (ODI) (20) data were collected at baseline, after one week, and after one, three, six, 12, 24, and 36 months of treatment. Baseline VAS and ODI scores were assessed face-to-face by the follow-up staff. After completing the evaluation of the baseline VAS and ODI scores, a follow-up schedule was developed for each patient to complete the follow-up on time. Postoperative patients were followed up face-to-face or through telephone by the follow-up staff.

### Evaluation of imaging outcomes

All cases of OVCF were classified according to the method of osteoporotic fracture classification (OF classification) by a radiological physician blinded to the grouping of this study (Table 1) (21). The OF classification was based on spinal X-radiography, computer tomography, and MRI, and was divided into five types according to the severity of the fracture. In addition, Schönrogge et al. found that this OVCF classification system showed good inter-observer reliability and significant intra-observer reliability (22). All patients identified as NVCF have been validated by MRI.

**Table 1:** The OF classification (21).

OF classification	Definition
Type 1	No vertebral deformation or compression was found in X ray and CT, but the presence of high intensity only in the MRI-short tau inversion recovery sequence, indicating vertebral body edema
Type 2	Involving only one endplate, with no or only minor posterior wall involved, less than one-fifth of the width of the posterior wall
Type 3	The distinct posterior wall involvement; more than one-fifth of the width of the posterior wall, or involvement of only one endplate
Type 4	Both endplates were involved, and a suspected severe deformity of the vertebral body, loss of integrity of the vertebral frame or vertebral body collapse or pincer-type fracture
Type 5	Injuries with distraction or rotation; Involving not only the anterior column but also posterior structures, such as, facet joints, ligaments or soft tissues, which could result in spinal instability

### Evaluation of patient satisfaction rate

This study assessed patient satisfaction rates at one week and one, three, six, 12, 24, and 36 months after treatment. The satisfaction rates were recorded by asking the patients whether they were “satisfied,” “very satisfied,” or “dissatisfied” with the outcomes of treatment (15). The reasons for patient satisfaction or dissatisfaction were counted, and the patient satisfaction rate (including satisfied and very satisfied patients) for each period was calculated.

### Treatment allocation

All patients were fully informed about the potential benefits and drawbacks of both PVP and CT. The treatment plan was finally decided by the patient after sufficient communication between the patient and attending physician.

CT group: Patients in the CT group received long-term anti-osteoporotic and symptomatic antalgic treatment.

PVP group: All patients underwent PVP surgery by local anesthesia. In addition to PVP surgery, patients in the PVP group underwent long-term anti-osteoporosis treatment after PVP surgery. The treatment plan for osteoporosis was determined by professional orthopedicians and endocrinologists.

Antalgic treatment: During the acute phase of the fracture (usually within one month after the fracture) in either the PVP or OVCF group, in addition to antiosteoporotic treatment, the physician administered analgesics such as nonsteroidal anti-inflammatory drugs or calcitonin to relieve the patient’s acute pain. In general, we will give patients 200 mg of oral celecoxib or a combination of salmon calcitonin during the acute phase of fractures.

### Statistical methods

Kolmogorov–Smirnov test was used to test the distribution of continuous variables. Student’s t test (mean ± standard deviation) or Mann–Whitney U test (median, lower quartile P25, upper quartile P75) was used to compare the age, BMI, BMD, back pain VAS, and ODI of the two groups, and chi-squared test was used to compare the sex, triggering factors of fractures, OF classification, site of fractures, treatment of anti-osteoporosis, satisfaction rates, and NVCF rates of the two groups. SPSS software (version 25; IBM Corp., Armonk, NY, USA) was used to analyze the data. The figures were drawn by the Graphpad Prism 8.0 (Graphpad Software Inc., San Diego. CA, USA). *p* < 0.05 was considered statistically significant for the differences between the two groups.

## Results

**Figure 1:**
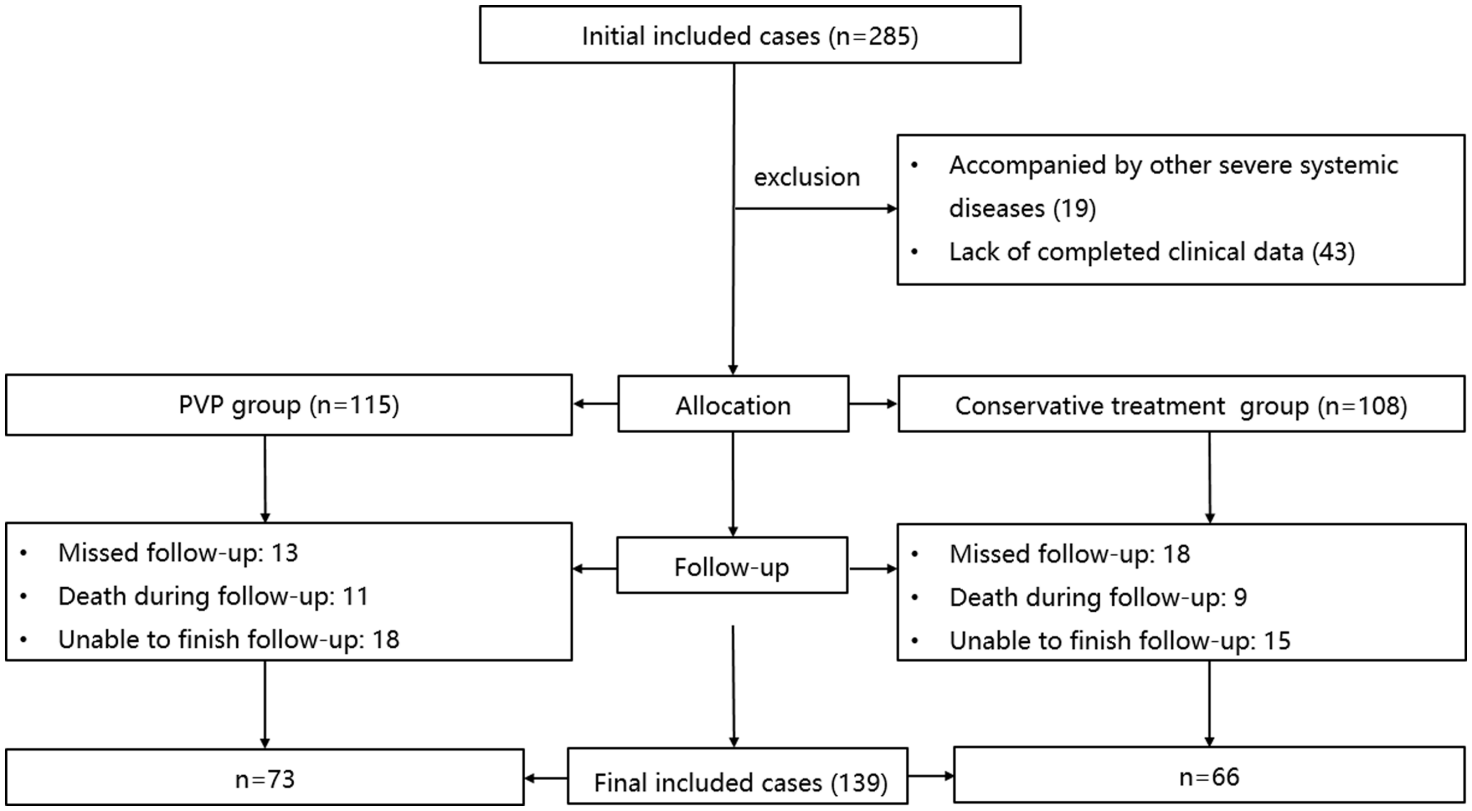
The inclusion and exclusion procedures of this study.

The inclusion and exclusion procedures are illustrated in Figure 1. Initially, 285 patients with OVCF were enrolled. After excluding 19 patients with other serious systemic diseases and 43 without complete data, 223 patients were included in the study. The PVP and CT groups included 115 and 108 patients, respectively. After excluding 31 patients who were lost to follow-up, 20 patients who died, and 33 patients who could not complete the follow-up, 139 patients were finally included in this study, including 73 and 66 patients in the PVP and CT groups, respectively.

### Demographics

Demographic characteristics are shown in Table 2. The study population included 54 men and 85 women. The average age, BMD, and BMI of patients are 73.6 ± 6.4 years, −3.5 ± 0.7, and 23.1 ± 2.5 kg/m^2^, respectively. There were no significant differences in sex, age, BMD, or BMI, between the two groups of patients. The duration of antalgic treatment in the PVP group during the acute phase of fractures was significantly lower than that in the CT group (3.6 ± 1.8 days *VS* 12.3 ± 3.6 days, *p* < 0.001). In addition, there was no significant difference in the triggering factors of fractures between the two groups. In the PVP group, 32 patients had hypertension, 13 patients had diabetes, and 2 patients had chronic obstructive pulmonary disease (COPD). In the CT group, 34 patients had hypertension, 12 patients had diabetes, 2 patient had rheumatoid arthritis, and 1 patient had COPD. There is no significant difference in the probability of comorbidities between the two groups of patients (PVP: 64.4% vs. CT: 74.2%, *p* = 0.209). The anti-osteoporosis treatment for the two groups are shown in Table 3. Calcium carbonate, vitamin D3, and bisphosphonates were the anti-osteoporosis regimens for most patients in both PVP and CT groups. Calcium carbonate, vitamin D3, and teriparatide were the second most frequently used anti-osteoporosis regimens in both groups. Due to poor patient compliance, 9 PVP and 6 CT patients received only oral Calcium carbonate and vitamin D3, respectively, and 3 PVP and 2 CT patients received only oral Calcium carbonate. In addition, one patient in the PVP group did not accept any anti-osteoporosis therapy after PVP surgery. This indicates that 17.8% (13/73) and 12.1% (8/66) of OVCF patients in the PVP and CT groups did not receive standardized anti-osteoporosis treatment, respectively.

**Table 2:** The demographics of the two groups.

Subgroup	PVP group (*N* = 73)	CT group (*N* = 66)	*p* value
Gender (male/female)	29/44	25/41	0.823
Age (years)	73.4 ± 5.9	73.8 ± 6.9	0.709
BMI (kg/m2)	23.2 ± 2.3	23.0 ± 2.8	0.551
BMD (T value)	−3.6 ± 0.7	−3.5 ± 0.6	0.612
Duration of antalgic treatment (day)	3.6 ± 1.8	12.3 ± 3.6	<0.001
Triggering factors of fractures			0.795
Falling	30	32	
Sudden load bearing	33	29	
Coughing	3	1	
Bending	3	2	
Traffic accident	1	0	
No obvious cause	3	2	

PVP, percutaneous vertebroplasty; CT, conservative treatment; BMD, bone mineral density; BMI, body mass index.

**Table 3:** The anti-osteoporosis treatment of the two groups.

Subgroup	PVP group (*N* = 73)	CT group (*N* = 66)	*p* value
5 anti-osteoporosis plans			0.598
Calcium carbonate, vitamin D3, bisphosphonates	38	31	
Calcium carbonate, vitamin D3, teriparatide	22	27	
Calcium carbonate, vitamin D3	9	6	
Calcium carbonate	3	2	
NA	1	0	

PVP, percutaneous vertebroplasty; CT, conservative treatment.

### Imaging outcomes

The imaging outcomes are shown in Table 4. In the PVP group, a total of 73 patients were accompanied by 80 segments of vertebral fractures. Among them, 7 patients had fractures of 2 or more segments, and 66 patients had single segment fractures. In the CT group, a total of 66 patients were accompanied by 71 segments of vertebral fractures. Among them, 4 patients had fractures of 2 or more segments, and 62 patients had single segment fractures. In the PVP group, there were 1 segment for type 1, 35 segments for type 2, 38 segments for type 3, 5 segments for type 4, and 1 segment for type 5. In the CT group, there were 2 segments for type 1, 37 segments for type 2, 29 segments for type 3, 2 segments for type 4, and 1 segment for type 5. There was no significant difference in the OF classification of fractures between the two groups of patients (*p* = 0.671). In the PVP group, 15 segments of OVCF occurred at the thoracic spine (T1-T11), 31 segments at the thoracolumbar spine (T12-L1), and 34 segments of OVCF occurred at the lumbar spine (L2-L5). In the CT group, 11 cases of OVCF occurred at the thoracic spine (T1-T11), 29 cases at the thoracolumbar segment (T12-L1), and 31 cases of OVCF occurred at the lumbar spine (L2-L5). There was no significant difference in the fracture site between the two groups of patients (*p* = 0.867).

**Table 4:** Comparison of imaging outcomes of the two groups.

Subgroup	PVP group (*N* = 73)	CT group (*N* = 66)	*p* value
OF classification (PVP group: 80 segments, CT group: 71 segments)			0.671
Type 1 (segment)	1	2	
Type 2 (segment)	35	37	
Type 3 (segment)	38	29	
Type 4 (segment)	5	2	
Type 5 (segment)	1	1	
Site of OVCF (PVP group: 80 segments, CT group: 71 segments)			0.867
Thoracic spine (T1-T11)	15	11	
Thoracolumbar spine (T12-L1)	31	29	
Lumbar spine (L2-L5)	34	31	

PVP, percutaneous vertebroplasty; CT, conservative treatment; OVCF, Osteoporotic vertebral compression fractures.

### Clinical outcomes

**Figure 2:**
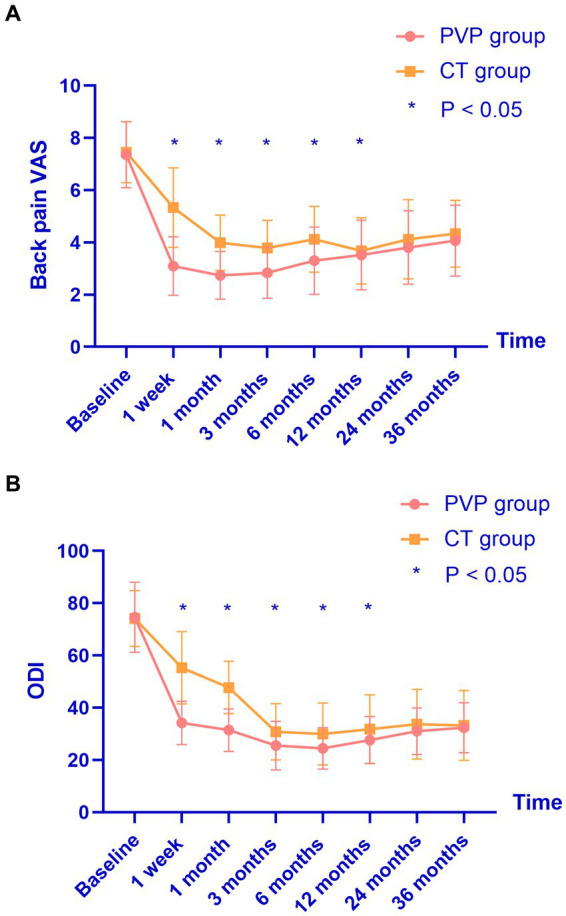
**(A,B)** The back pain VAS and ODI scores of the CT group were significantly higher than those of the PVP group at one week and one, three, six, and 12 months after treatment. However, they showed no significant differences between the two groups at 24 and 36 months. VAS, visual analogue scale; ODI, oswestry disability index; PVP, percutaneous vertebroplasty; CT, conservative treatment.

**Figure 3:**
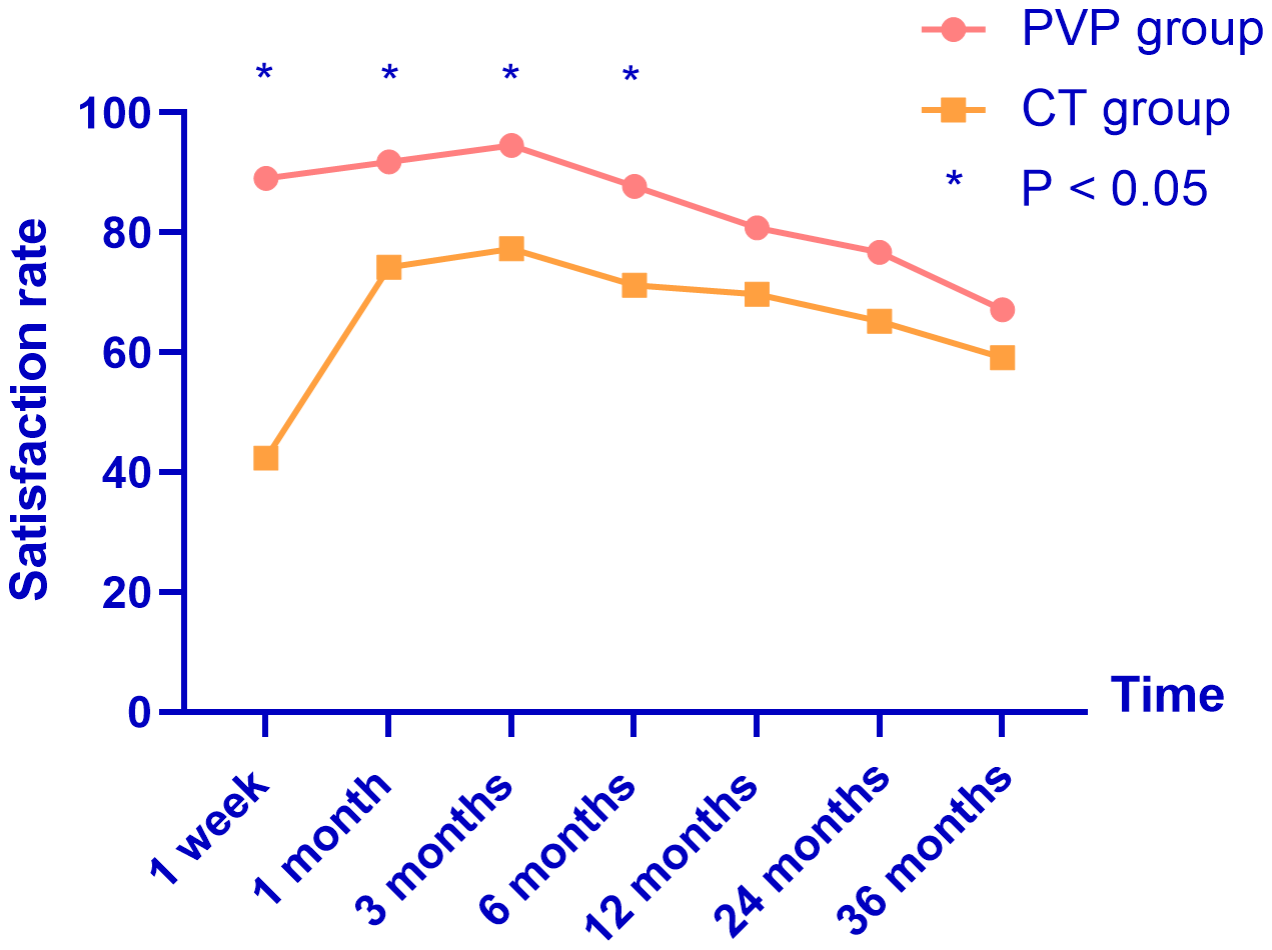
The satisfaction rates of both groups reached their highest point three months after treatment, and gradually decreased thereafter. PVP, percutaneous vertebroplasty; CT, conservative treatment.

The clinical outcomes are shown in Table 5 and Figure 2. There were no significant differences in the baseline back pain VAS and ODI scores between the two groups. Both PVP and CT significantly improved back pain VAS and ODI scores. The back pain VAS and ODI scores of the CT group were significantly higher than those of the PVP group at one week and one, three, six, and 12 months after treatment. Subsequently, the back pain VAS and ODI scores showed no significant differences between the two groups at 24 and 36 months. Figures 2, 3 show the gradual convergence of back pain VAS and ODI scores between the PVP and CT groups with an increase in follow-up duration.

**Table 5:** Comparison of BP VAS and ODI of the two groups.

Subgroup	PVP group (*N* = 73)	CT group (*N* = 66)	*p* value
Baseline PB VAS	7.4 ± 1.3	7.4 ± 1.2	0.635
1 week BP VAS	3.1 ± 1.1	5.3 ± 1.5	<0.001
1 month BP VAS	2.7 ± 0.9	4.0 ± 1.1	<0.001
3 months BP VAS	2.8 ± 1.0	3.8 ± 1.1	<0.001
6 months BP VAS	3.3 ± 1.3	4.1 ± 1.3	<0.001
12 months BP VAS	3.5 ± 1.3	4.0 ± 1.2	0.022
24 months BP VAS	3.8 ± 1.4	4.1 ± 1.5	0.254
36 months BP VAS	4.1 ± 1.4	4.3 ± 1.3	0.240
Baseline ODI	73.33 (66.67,84.44)	75.56 (66.67,82.22)	0.817
1 week BP DOI	35.56 (28.89,40.00)	53.33 (44.44,62.22)	<0.001
1 month BP ODI	31.2 ± 8.1	47.7 ± 10.0	<0.001
3 months BP ODI	25.5 ± 9.3	30.8 ± 10.8	0.002
6 months BP DOI	24.44 (20.00,28.89)	26.67 (22.22,37.78)	0.002
12 months BP DOI	27.67 (22.22,34.45)	31.11 (20.00,42.22)	0.032
24 months BP DOI	31.11 (24.44,37.78)	35.56 (22.22,42.78)	0.164
36 months BP DOI	33.33 (25.56,37.78)	32.22 (21.94,42.78)	0.662

BP, back pain; ODI, oswestry disability index; VAS, visual analogue scale; PVP, percutaneous vertebroplasty; CT, conservative treatment.

### Satisfaction rates

The patient satisfaction rates are shown in Table 6 and Figure 3. The satisfaction rates in the PVP group were significantly higher than those in the CT group at one week and one, three, and six months after treatment. However, although the rate of satisfaction was slightly higher in the PVP group than in the CT group at 12, 24, and 36 months after treatment, the difference was not significant. This indicates that after 12 months, the satisfaction rates of the two groups of patients with the treatment gradually tends to be close.

**Table 6:** Comparison of satisfaction rate of the two groups.

Subgroup	PVP group (*N* = 73)	CT group (*N* = 66)	*p* value
1 week satisfaction rate	89.0% (65/73)	42.2% (28/66)	<0.001
1 month satisfaction rate	91.8% (67/73)	74.2% (49/66)	0.005
3 months satisfaction rate	94.5% (69/73)	77.3% (51/66)	0.003
6 months satisfaction rate	87.7% (64/73)	71.2% (47/66)	0.016
12 months satisfaction rate	80.8% (59/73)	69.7% (46/66)	0.128
24 months satisfaction rate	76.7% (56/73)	65.2% (43/66)	0.133
36 months satisfaction rate	67.1% (49/73)	59.1% (39/66)	0.326

PVP, percutaneous vertebroplasty; CT, conservative treatment.

### NVCF rates

**Figure 4:**
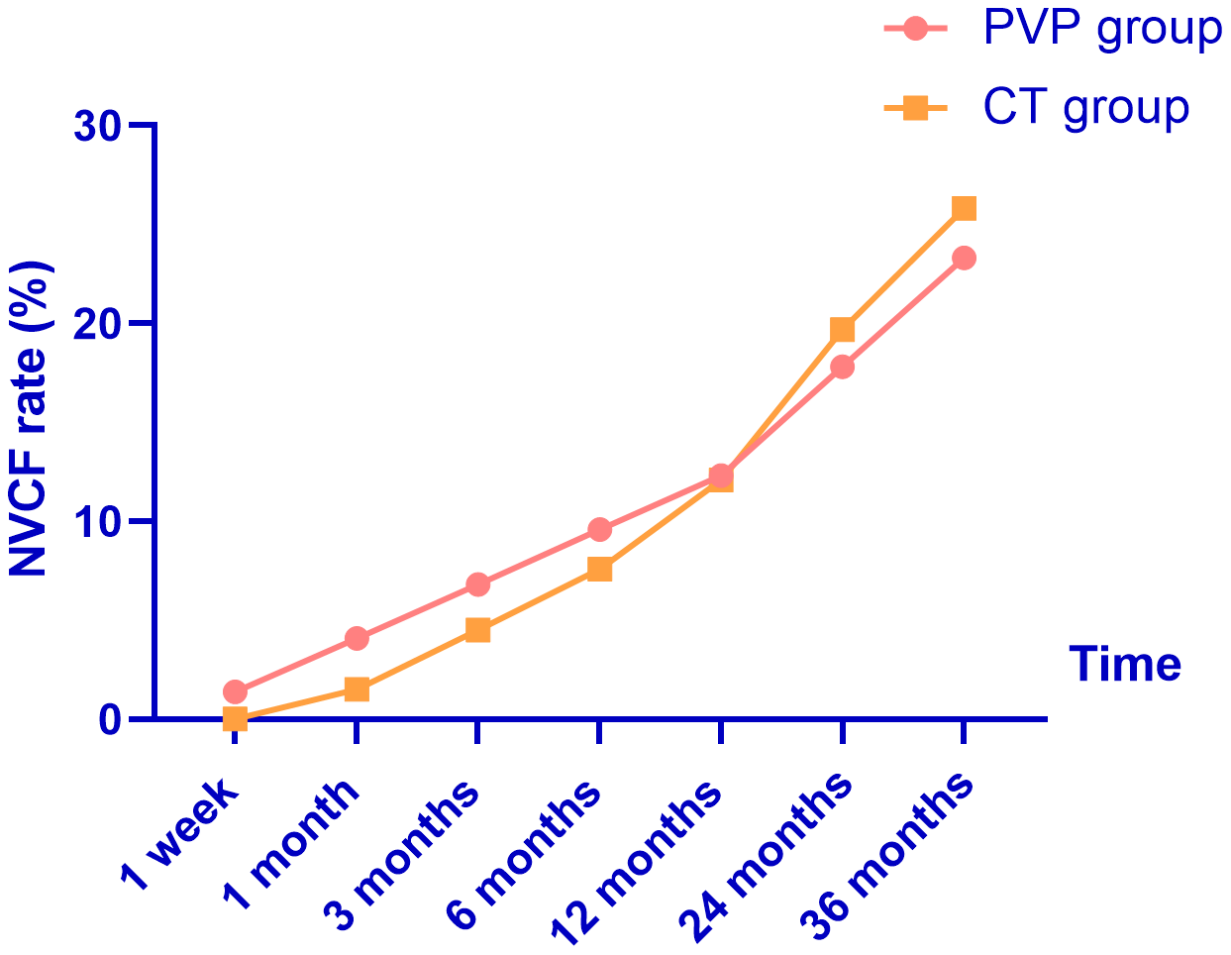
The probability of NVCF gradually increases with the extension of follow-up time. NVCF, new vertebral compression fractures; PVP, percutaneous vertebroplasty; CT, conservative treatment.

The NVCF rates are shown in Table 7 and Figure 4. In this study, the NVCF rate within 36 months of the initial OVCF was 24.5% (34/139). Although the NVCF rate in the PVP group was slightly lower than that in the CT group, the difference between the two groups was not statistically significant (23.3% vs. 25.8%, *p* = 0.735). It should be noted that the treatment of patients with NVCF is not solely determined by the physician. After the physician fully informs the patient of the possible advantages and disadvantages of PVP surgery or CT, the choice of NVCF patients to continue treatment is up to the patient. Among the 17 patients with NVCF in the PVP group, 11 patients were treated with re-PVP surgery, and 6 patients were treated with CT including bed rest, antalgic drugs and anti-osteoporosis drugs. Among the 17 patients with NVCF in the CT group, 13 patients were treated with re-PVP surgery, and 4 patients were treated with CT including bed rest, antalgic drugs and anti-osteoporosis drugs.

**Table 7:** Comparison of NVCF rate of the two groups.

Subgroup	PVP group (*N* = 73)	CT group (*N* = 66)	*p* value
1 week	1	0	
1 month	2	1	
3 months	2	2	
6 months	2	2	
12 months	2	3	
24 months	4	5	
36 months	4	4	
sum up	17 (23.3%)	17 (25.8%)	0.735

NVCF, new vertebral compression fractures; PVP, percutaneous vertebroplasty; CT, conservative treatment.

## Discussion

In this study, we compared the clinical outcomes within 36 months of PVP and CT in patients with OVCF. We found that both treatment methods improved the patients’ back pain VAS and ODI scores. The back pain VAS and ODI scores of the CT group were significantly higher than those of the PVP group at one week and one, three, six, and 12 months after treatment. The satisfaction rate in the PVP group was significantly higher than that in the CT group at one week and one, three, and six months after treatment. Subsequently, back pain VAS and ODI scores showed no significant differences between the two groups at 24 and 36 months. In addition, there was no significant difference in treatment satisfaction between the two groups at 12, 24, and 36 months. We did not find a significant difference in the probability of NVCF between the two groups within 36 months of treatment.

Whether OVCF should be treated surgically or conservatively was controversial (11–18). A randomized clinical study by Rousing et al. compared the clinical outcomes within three months of PVP and CT in patients with painful acute (< 2 weeks) or subacute (between 2 and 8 weeks) OVCF (41 women). They found that pain relief within 12 to 24 h after PVP surgery was significantly higher than that of CT, while pain improvement between the two groups at three months showed no significant difference (11). However, a subsequent open-label randomized trial involving 202 patients by Klazen et al. found that patients with acute OVCF and persistent pain in the PVP surgery achieved better pain improvement than CT and showed maintenance of results for at least one year (12). Subsequently, a prospective study including 259 patients by Lee et al. found that PVP surgery alone had better clinical outcomes than CT within one month, whereas both groups showed similar clinical outcomes at one year. They found that the risk factors for failure of three weeks by CT were older age (age > 78.5 years), overweight (BMI > 25.5 kg/m^2^), severe osteoporosis (t score < −2.95), and larger collapse rates (> 28.5%). The authors suggested that PVP should not be recommended in patients with OVCF who have no risk factors for failure of CT (13). In addition, Blasco et al. found that PVP can achieve better pain improvement at two months than CT, but there was no significant difference in pain improvement or quality of life between the two groups after one year (14). However, a recent randomized controlled trial (56 patients in the PVP group and 51 in the CT group) by Yang et al. found that PVP could achieve better pain relief and quality of life at one week and one, three, six, and 12 months after treatment (15). Additionally, a retrospective study (over two-year follow-up) by Yi et al. found that PVP can improve pain better than CT at one, two, and three weeks, and six months after treatment, whereas no significant difference was found between the two groups after one and two years (16). The follow-up duration in this study is longer than that in previous studies because we investigated the clinical outcomes of patients with OVCF within 36 months after PVP and CT. We found better pain improvement and quality of life (VAS and ODI) in PVP group than those in CT group at one week and one, three, six, and 12 months after treatment. However, at 24 and 36 months after treatment, no significant difference was found in the VAS and ODI scores between the two groups. This indicates that the advantages of PVP decrease gradually, leading to a preference for CT.

Yang et al. found that the patient satisfaction rate after one year of PVP was significantly higher than that after CT (73.2% vs. 58.8%) (15). Similar to the study by Yang et al., the satisfaction rate in the PVP group was significantly higher than that in the CT group at one week and one, three, and six months after treatment. However, the satisfaction rates of the two groups gradually tended to be consistent at 12, 24, and 36 months, although the NVCF rate in the PVP group was slightly lower than that in the CT group. This indicates that in the medium-to-long term, PVP does not result in significantly higher patient satisfaction rates than CT.

NVCF after OVCF is common in clinical practice. According to previous reports, the incidence of NVCF after OVCF ranges from 9.3 to 38.4% (23–33). Although the conclusions vary, most studies and systematic reviews have found no significant differences in the probability of NVCF between PVP and CT (23–33). A randomized controlled trial including 363 patients with OVCF by Yi et al. found that PVP or percutaneous kyphoplasty was not associated with an increased risk of NVCF compared to CT (34–37). Additionally, a recent systematic review by Xie et al. found no significant differences in the rate of adjacent vertebral fractures between the PVP and CT groups (18). In this study, similar to Yi’s and Xie’s studies, we did not find a significant difference in the incidence of NVCF between the two groups of patients with OVCF at 36 months. This indicates that PVP does not increase the risk of NVCF in the long term. In addition, in this series of patients, there are some cases of sudden low back pain after PVP or CT. However, they did not perform definitive examinations (MRI) to confirm the diagnosis of NVCF. Therefore, the NVCF probability of patients in this study may be underestimated. Thus, considering the high incidence of NVCF, standardized postoperative anti-osteoporosis treatment is recommended to reduce the incidence of NVCF (38). In addition, a possible reason for the high NVCF rate in this study is that a considerable number of patients did not undergo strict anti-osteoporosis treatment after PVP (17.8%) or CT (12.1%).

This study has few limitations. First, this was a retrospective study and may have unavoidable biases. In addition, the number of cases in this study was limited and the patient dropout rate was relatively high. Finally, this study was conducted at a single center, and further exploration is needed to determine whether the conclusions drawn are equally applicable to other institutions.

## Conclusion

In conclusion, the clinical outcomes within 12 months after PVP and the patient satisfaction rate within six months after PVP were significantly better than those after CT. However, from 12 to 36 months after treatment, this advantage due to PVP decreased gradually. Compared to CT, the long-term effect of PVP on OVCF should not be overestimated.

## Data availability statement

The raw data supporting the conclusions of this article will be made available by the authors, without undue reservation.

## Ethics statement

The studies involving humans were approved by the ethics committee of Xiangyang No.1 People’s Hospital. The studies were conducted in accordance with the local legislation and institutional requirements. The ethics committee/institutional review board waived the requirement of written informed consent for participation from the participants or the participants’ legal guardians/next of kin because this is a retrospective study and it is difficult to obtain signed consent from all participants. Therefore, we have applied to the ethics committee for exemption from signing informed consent forms. But this study received verbal consent from the participants.

## Author contributions

ZC: Investigation, Writing – original draft, Data curation, Formal analysis. LX: Investigation, Data curation, Writing – review & editing. LS: Writing – review & editing, Methodology, Resources, Software. HC: Writing – review & editing, Investigation, Supervision, Project administration. MN: Conceptualization, Writing – review & editing, Supervision, Validation.

## References

[1] Sfeir JG Drake MT Khosla S Farr JN. Skeletal Aging. Mayo Clin Proc. (2022) 97:1194–208. doi: 10.1016/j.mayocp.2022.03.011, PMID: 35662432 PMC9179169

[2] Lane NE. Epidemiology, etiology, and diagnosis of osteoporosis. Am J Obstet Gynecol. (2006) 194:S3–S11. doi: 10.1016/j.ajog.2005.08.047 16448873

[3] Wright NC Saag KG Dawson-Hughes B Khosla S Siris ES. The impact of the new National Bone Health Alliance (NBHA) diagnostic criteria on the prevalence of osteoporosis in the USA. Osteoporos Int. (2017) 28:1225–32. doi: 10.1007/s00198-016-3865-3, PMID: 27966104

[4] Zhu H Fang J Luo X Yu W Zhao Y Li X. A survey of bone mineral density of healthy Han adults in China. Osteoporos Int. (2010) 21:765–72. doi: 10.1007/s00198-009-1010-2 19597908

[5] Yu F Xia W. The epidemiology of osteoporosis, associated fragility fractures, and management gap in China. Arch Osteoporos. (2019) 14:32. doi: 10.1007/s11657-018-0549-y, PMID: 30848398

[6] Kwok AW Gong JS Wang YX Leung JC Kwok T Griffith JF. Prevalence and risk factors of radiographic vertebral fractures in elderly Chinese men and women: results of Mr. OS (Hong Kong) and Ms. OS (Hong Kong) studies. Osteoporosis Int. (2013) 24:877–85. doi: 10.1007/s00198-012-2040-8, PMID: 22707064

[7] Luthman S Widén J Borgström F. Appropriateness criteria for treatment of osteoporotic vertebral compression fractures. Osteoporos Int. (2018) 29:793–804. doi: 10.1007/s00198-017-4348-x 29260290

[8] Choi SH Kim DY Koo JW Lee SG Jeong SY Kang CN. Incidence and management trends of osteoporotic vertebral compression fractures in South Korea: a Nationwide population-based study. Asian Spine J. (2020) 14:220–8. doi: 10.31616/asj.2019.0051, PMID: 31668050 PMC7113475

[9] Cummings SR Melton LJ. Epidemiology and outcomes of osteoporotic fractures. Lancet. (2002) 359:1761–7. doi: 10.1016/S0140-6736(02)08657-9 12049882

[10] Yang YS Tsou YS Lo WC Chiang YH Lin JH. Teriparatide associated with fewer Refractures and higher Body Heights of cemented vertebrae after Vertebroplasty: a matched cohort study. Sci Rep. (2020) 10:6005. doi: 10.1038/s41598-020-62869-0, PMID: 32265470 PMC7138790

[11] Rousing R Andersen MO Jespersen SM Thomsen K Lauritsen J. Percutaneous vertebroplasty compared to conservative treatment in patients with painful acute or subacute osteoporotic vertebral fractures: three-months follow-up in a clinical randomized study. Spine (Phila Pa 1976). (2009) 34:1349–54. doi: 10.1097/BRS.0b013e3181a4e628 19478654

[12] Klazen CA Lohle PN de Vries J Jansen FH Tielbeek AV Blonk MC. Vertebroplasty versus conservative treatment in acute osteoporotic vertebral compression fractures (Vertos II): an open-label randomised trial. Lancet. (2010) 376:1085–92. doi: 10.1016/S0140-6736(10)60954-3 20701962

[13] Lee HM Park SY Lee SH Suh SW Hong JY. Comparative analysis of clinical outcomes in patients with osteoporotic vertebral compression fractures (OVCFs): conservative treatment versus balloon kyphoplasty. Spine J. (2012) 12:998–1005. doi: 10.1016/j.spinee.2012.08.024, PMID: 23026068

[14] Blasco J Martinez-Ferrer A Macho J San Roman L Pomés J Carrasco J. Effect of vertebroplasty on pain relief, quality of life, and the incidence of new vertebral fractures: a 12-month randomized follow-up, controlled trial. J Bone Miner Res. (2012) 27:1159–66. doi: 10.1002/jbmr.1564, PMID: 22513649

[15] Yang EZ Xu JG Huang GZ Xiao WZ Liu XK Zeng BF. Percutaneous Vertebroplasty versus conservative treatment in aged patients with acute osteoporotic vertebral compression fractures: a prospective randomized controlled clinical study. Spine (Phila Pa 1976). (2016) 41:653–60. doi: 10.1097/BRS.0000000000001298 26630417

[16] Yi HJ Jeong JH Im SB Lee JK. Percutaneous vertebroplasty versus conservative treatment for one level thoracolumbar osteoporotic compression fracture: results of an over 2-year follow-up. Pain Physician. (2016) 19:E743–50. PMID: 27389117

[17] Firanescu CE de Vries J Lodder P Venmans A Schoemaker MC Smeets AJ. Vertebroplasty versus sham procedure for painful acute osteoporotic vertebral compression fractures (VERTOS IV): randomised sham controlled clinical trial. BMJ. (2018) 361:k1551. doi: 10.1136/bmj.k1551 29743284 PMC5941218

[18] Xie L Zhao ZG Zhang SJ Hu YB. Percutaneous vertebroplasty versus conservative treatment for osteoporotic vertebral compression fractures: an updated meta-analysis of prospective randomized controlled trials. Int J Surg. (2017) 47:25–32. doi: 10.1016/j.ijsu.2017.09.021, PMID: 28939236

[19] Chapman JR Norvell DC Hermsmeyer JT Bransford RJ DeVine J McGirt MJ. Evaluating common outcomes for measuring treatment success for chronic low back pain. Spine (Phila Pa 1976). (2011) 36:S54–68. doi: 10.1097/BRS.0b013e31822ef74d 21952190

[20] Fairbank JC Pynsent PB. The Oswestry disability index. Spine (Phila Pa 1976). (2000) 25:2940–53. doi: 10.1097/00007632-200011150-00017 11074683

[21] Schnake KJ Blattert TR Hahn P Franck A Hartmann F Ullrich B. Classification of osteoporotic thoracolumbar spine fractures: recommendations of the spine section of the German Society for Orthopaedics and Trauma (DGOU). Global Spine J. (2018) 8:46S–9S. doi: 10.1177/2192568217717972, PMID: 30210960 PMC6130101

[22] Schönrogge M Lahodski V Otto R Adolf D Damm R Sitte-Zöllner A. Inter-and intraobserver reliabilities and critical analysis of the osteoporotic fracture classification of osteoporotic vertebral body fractures. Eur Spine J. (2022) 31:2431–8. doi: 10.1007/s00586-022-07201-2, PMID: 35378632

[23] Rho YJ Choe WJ Chun YI. Risk factors predicting the new symptomatic vertebral compression fractures after percutaneous vertebroplasty or kyphoplasty. Eur Spine J. (2012) 21:905–11. doi: 10.1007/s00586-011-2099-5, PMID: 22160212 PMC3337901

[24] Ren HL Jiang JM Chen JT Wang JX. Risk factors of new symptomatic vertebral compression fractures in osteoporotic patients undergone percutaneous vertebroplasty. Eur Spine J. (2015) 24:750–8. doi: 10.1007/s00586-015-3786-4, PMID: 25645589

[25] Yang S Liu Y Yang H Zou J. Risk factors and correlation of secondary adjacent vertebral compression fracture in percutaneous kyphoplasty. Int J Surg. (2016) 36:138–42. doi: 10.1016/j.ijsu.2016.10.030, PMID: 27777054

[26] Li HM Zhang RJ Gao H Jia CY Zhang JX Dong FL. New vertebral fractures after osteoporotic vertebral compression fracture between balloon kyphoplasty and nonsurgical treatment PRISMA. Medicine (Baltimore). (2018) 97:e12666. doi: 10.1097/MD.0000000000012666, PMID: 30290650 PMC6200511

[27] Lee BG Choi JH Kim DY Choi WR Lee SG Kang CN. Risk factors for newly developed osteoporotic vertebral compression fractures following treatment for osteoporotic vertebral compression fractures. Spine J. (2019) 19:301–5. doi: 10.1016/j.spinee.2018.06.347, PMID: 29959099

[28] Chen C Fan P Xie X Wang Y. Risk factors for cement leakage and adjacent vertebral fractures in Kyphoplasty for osteoporotic vertebral fractures. Clin Spine Surg. (2020) 33:E251–5. doi: 10.1097/BSD.0000000000000928, PMID: 32011354

[29] Zhang ZL Yang JS Hao DJ Liu TJ Jing QM. Risk factors for new vertebral fracture after percutaneous Vertebroplasty for osteoporotic vertebral compression fractures. Clin Interv Aging. (2021) 16:1193–200. doi: 10.2147/CIA.S312623, PMID: 34188462 PMC8235945

[30] Chen Z Song C Chen M Li H Ye Y Liu W. What are risk factors for subsequent fracture after vertebral augmentation in patients with thoracolumbar osteoporotic vertebral fractures. BMC Musculoskelet Disord. (2021) 22:1040. doi: 10.1186/s12891-021-04946-7, PMID: 34903222 PMC8670201

[31] Park JS Park YS. Survival analysis and risk factors of new vertebral fracture after vertebroplasty for osteoporotic vertebral compression fracture. Spine J. (2021) 21:1355–61. doi: 10.1016/j.spinee.2021.04.022, PMID: 33971326

[32] Ji C Rong Y Wang J Yu S Yin G Fan J. Risk factors for Refracture following primary osteoporotic vertebral compression fractures. Pain Physician. (2021) 24:E335–40. doi: 10.36076/ppj.2021/24/E335, PMID: 33988955

[33] Cheng Y Cheng X Wu H. Risk factors of new vertebral compression fracture after percutaneous vertebroplasty or percutaneous kyphoplasty. Front Endocrinol (Lausanne). (2022) 13:964578. doi: 10.3389/fendo.2022.964578, PMID: 36120447 PMC9470857

[34] Chen LX Li YL Ning GZ Li Y Wu QL Guo JX. Comparative efficacy and tolerability of three treatments in old people with osteoporotic vertebral compression fracture: a network meta-analysis and systematic review. PLoS One. (2015) 10:e0123153. doi: 10.1371/journal.pone.0123153, PMID: 25874802 PMC4395314

[35] Yi X Lu H Tian F Wang Y Li C Liu H. Recompression in new levels after percutaneous vertebroplasty and kyphoplasty compared with conservative treatment. Arch Orthop Trauma Surg. (2014) 134:21–30. doi: 10.1007/s00402-013-1886-3, PMID: 24287674 PMC3889698

[36] Yang W Song J Liang M Cui H Chen H Yang J. Functional outcomes and new vertebral fractures in percutaneous Vertebroplasty and conservative treatment of acute symptomatic osteoporotic vertebral compression fractures. World Neurosurg. (2019) 131:e346–52. doi: 10.1016/j.wneu.2019.07.153, PMID: 31356973

[37] Zhang H Xu C Zhang T Gao Z Zhang T. Does percutaneous Vertebroplasty or balloon Kyphoplasty for osteoporotic vertebral compression fractures increase the incidence of new vertebral fractures? A Meta-Analysis. Pain Physician. (2017) 20:E13–28. doi: 10.36076/ppj.2017.1.E13, PMID: 28072794

[38] Qin J Zhong W Quan Z. The surgical management trends of osteoporotic vertebral compression fractures: 5-year experience in one institution. Sci Rep. (2022) 12:18040. doi: 10.1038/s41598-022-23106-y, PMID: 36302942 PMC9613630

